# The effects of non-nutritive sweeteners on energy and macronutrients intake in adults: a grade-assessed systematic review and meta-analyses of randomized controlled trials

**DOI:** 10.3389/fnut.2024.1475962

**Published:** 2024-11-13

**Authors:** Kimia Rostampour, Fatemeh Moghtaderi, AmirHossein Najafi, Behnaz Seyedjafari, Amin Salehi-Abargouei

**Affiliations:** ^1^Student Research Committee, Shahid Sadoughi University of Medical Sciences, Yazd, Iran; ^2^Research Center for Food Hygiene and Safety, School of Public Health, Shahid Sadoughi University of Medical Sciences, Yazd, Iran; ^3^Department of Nutrition, School of Public Health, Shahid Sadoughi University of Medical Sciences, Yazd, Iran; ^4^Yazd Cardiovascular Research Center, Non-communicable Diseases Research Institute, Shahid Sadoughi University of Medical Sciences, Yazd, Iran

**Keywords:** non-nutritive sweeteners, meta-analysis, nutrients intake, total energy intake, carbohydrate intake

## Abstract

**Objectives:**

The effect of non-nutritive sweeteners (NNSs) on long-term satiety is not well understood. This systematic review and meta-analysis were performed to investigate the effect of NNSs on long-term total energy and macronutrients intake.

**Methods:**

Online databases including Scopus, PubMed, ISI Web of Science, and Google Scholar were searched up to September 2024 to find relevant randomized control trials (RCTs). A random effects model was used for estimating the overall effects.

**Results:**

The results showed a reducing effect of NNSs consumption vs. sugar on total energy intake [total energy intake change = −175.26 kcal/day, 95% confidence interval (CI): −296.47 to −54.06, I^2^ = 61.19%] and carbohydrate intake [Hedges’ g = −0.35, 95% CI: −0.63 to −0.06, I^2^ = 58.99%]. While, NNSs intake vs. water was not associated with significant change in total energy intake [total energy intake change = 29.94 kcal/day, 95% CI: −70.37 to 130.24, I^2^ = 34.98%] and carbohydrate intake [Hedges’ g = 0.28, 95% CI: −0.02 to 0.58, I^2^ = 65.26%]. The Consumption of NNSs compared to the either sugar or water did not have a significant effect on fat intake [Hedges’ g _sugar_ = 0.08, 95% CI: −0.10 to 0.26, I^2^ = 8.73%/ fat intake change _water_ = 0.20 g/day, 95% CI: −3.48 to 3.88, I^2^ = 0%] and Protein intake [Hedges’ g _sugar_ = 0.16, 95% CI: −0.11 to 0.42, I_^2^_ = 50.83%/Hedges’ g _water_ = 0.00, 95% CI: −0.15 to 0.16, I^2^ = 0%].

**Conclusion:**

In summary, our findings suggest that NNSs consumption may be effective in reducing total energy and carbohydrate intake compared to sugar.

**Systematic Review Registration:**

https://www.crd.york.ac.uk/prospero/display_record.php?RecordID=432816, CRD42023432816.

## 1 Introduction

Obesity is a prominent global health concern (1), affecting not only low- and middle-income countries but also high-income countries (2, 3). Based on reports from the World Health Organization (WHO), global obesity rates have experienced a threefold increase since 1975 (4). Obesity leads to inflammatory conditions in adipose tissue, causing metabolic diseases including hypertension, cardiovascular diseases, insulin resistance, and cancers, which are attributable for the death of 17 million people each year worldwide (1, 5).

Diets rich in energy-dense foods and beverages, such as sugar-sweetened beverages, has been linked to an increased risk of obesity and chronic diseases by contributing to enhanced energy intake (6, 7). Moreover, high consumption of free sugar might lead to less intake of essential micronutrients from healthy food choices, reducing diet quality and thus increasing the risk of nutrient deficiencies (8–10). Acknowledging the adverse effects of high sugar consumption, the World Health Organization limits the consumption of sugars to 10% of daily energy intake (11). Due to the disadvantages of sugar-sweetened beverages (SSBs) as a major contributor to the consumption of added sugars, non-nutritive sweeteners (NNSs) were introduced as an alternative. According to the published literature, the consumption of non-nutritive sweeteners has increased in recent years (12, 13). Aspartame, acesulfame-K, neotame, saccharin, sucralose and advantame are approved by the United States Food and Drug Administration (FDA) are used in a wide range of foods and beverages. The FDA guarantees their safety up to acceptable daily intake levels (14). Additionally, stevia has approval of Codex commission which consists of the WHO and the Food Agriculture Organization (FAO) (15).

Although theoretically NNSs should reduce energy and carbohydrate intake, there are controversial results regarding their effect on satiety and energy balance as marker of long-term satiety (16–18). Some RCTs have suggested a reducing effect of diet beverage consumption on energy intake (17, 19). However, Orku et al. reported that NNS consumption was not significantly related to energy and macronutrient intake (16). It has been shown that replacing sugar with artificial sweeteners leads to a decrease in energy and sugar intake in healthy, obese, and overweight people (20). However, there is a hypothesis that the consumption of non-nutritive sweeteners causes disturbances in appetite control (21, 22), and there is a concern that the intake of these sweeteners increases the desire for sweet and energy-containing foods (23, 24).

As far as we are aware, no comprehensive research on the effects of these sweeteners on macronutrients intake has been conducted so far. Therefore, the present study aimed to systematically review the effects of NNSs on total energy and macronutrient intake, with a subsequent meta-analysis to confirm the findings.

## 2 Materials and methods

This study was done as part of a large project aimed at investigating the effects of non-nutritive sweeteners on various aspects of health in adults. The protocol for the main study was registered in the prospective register of systematic reviews (PROSPERO) database in June 2023 (registration code: CRD42023432816). We followed the Preferred Reporting Items for Systematic Reviews and Meta-Analysis (PRISMA) guideline (25) to report the current study. The ethics committee of Shahid Sadoughi University of Medical Sciences, Yazd, Iran, approved the protocol of the current study (ethical approval code: IR.SSU.SPH.REC.1402.119).

### 2.1 Study selection criteria

The search strategy was carried out in online databases including PubMed, ISI Web of Science, Scopus, and Google Scholar up to September 2024 using two sets of the following keywords: (1) “Non Nutritive Sweeteners,” “Non Nutritive Sweeteners,” “artificial sweeteners,” “Artificially Sweetened Soda,” “non-caloric sweeteners,” “non caloric sweeteners,” “zero-calorie sweetener,” “high-intensity sweetener,” “sugar substitute,” “Low-calorie sweeteners,” “artificial sugar,” “Sweetening Agents,” Aspartame, Stevia, Saccharin, acetosulfame, “acesulfame K,” “acesulfame potassium,” NutraSweet, Splenda, Cyclamates, “Steviol glycosides,” “rebaudioside A,” newtame, “sugar twin,” “monk fruit,” “rebaudioside D,” and stevioside. (2) intervention, trial, randomized, random, randomly, placebo, assignment, “clinical trial,” RCT, “Clinical Trials as Topic,” cross-over, parallel. There were no language or other limitations. To find possible new articles, the reference lists of the included articles were thoroughly checked (Supplementary Table 1).

Studies with the following criteria were included in the current meta-analysis: (1) Randomized controlled trials (RCTs) with parallel or crossover design, (2) studies with a duration of at least 4 weeks, (3) studies involving individuals aged 18 and older. Trials were excluded if they contained sugar alcohols, were performed on children and adolescents, had a duration of less than 4 weeks, did not provide sufficient data, were animal or *in-vitro* studies. If there were several articles on a data set, the most complete one was considered. Additionally, to extract data from articles with an additional arm, they were considered as a separate study.

### 2.2 Data extraction

Two investigators (KR, AHN) independently extracted the data. The accuracy of the extracted data was checked by two other researchers (FM, BS), and any discrepancies were resolved under the supervision of another investigator (ASA). The extracted data included the following items: authors’ names, publication year, type of NNSs and control, sample size, population characteristics (age, gender, body mass index, health status of the participants), type of study (parallel or cross-over), duration of intervention, and mean and standard deviation (SD) values of total energy, sugar, fiber, and macronutrient intake.

### 2.3 Risk of bias assessment

Included studies were evaluated using Version 2 of the Cochrane risk-of-bias tool for randomized trials (RoB2) (26). The following domains were assessed for each study: randomization process, deviations from the intended interventions (effect of assignment to intervention), deviations from the intended interventions (effect of adhering to intervention), missing outcome data, inappropriate measurement of the outcome, and selection of the reported results. Each domain received high, low or some concerns. Overall, each study was categorized as low risk (low risk of bias for all items), some concerns (one or more items with some concerns), or high risk (high risk of bias for one or more items). The assessment was initially conducted by one author (KR), with a second author verifying the risk assessment (FM).

### 2.4 Statistical analyses

Mean change from baseline and its standard deviation (SD) in total energy and nutrient intake were calculated for both the intervention and comparison groups. The difference in mean change between the intervention and control groups and its corresponding SD was then determined (27, 28). A correlation coefficient (R) of 0.50 was assumed for this calculation. Notably, using a correlation coefficient of 0.1 and 0.9 did not yield significantly different results. Subsequently, we calculated the bias-corrected standardized mean difference (hedges’ g) for the meta-analyses of all nutrients except for the total energy intake which was reported as Kcal/day, and for the effects of NNSs on fiber intake vs water, on sugar intake vs water, and on fat intake vs water which were reported as g/day. Indeed, all analyses were performed separately based on the type of control group intervention (sugar/water). For the effect of non-nutritive sweeteners consumption on sugar intake based on control group intervention (sugar/water), in NNSs vs. sugar subgroup, we calculated the bias-corrected standardized mean difference (hedges’ g) for the meta-analyses of sugar intake as the final effect size due to the varying units reported in different studies, which could not be converted into the same unit. But, for the effect of non-nutritive sweeteners consumption on sugar intake in NNSs vs. water, included studies reported same unit, therefore, we calculated the WMD for the meta-analyses as the effect size. An inverse variance random effects model was selected to calculate pooled estimates. Cochran Q test and I-squared (I^2^) were used to measure heterogeneity across included studies (29). An I^2^ > 50% or *p* < 0.05 for Q test indicated significant heterogeneity between studies. It should be noted that all analyses were performed separately based on the type of control group intervention (sugar/water). Subgroup analysis was conducted based on the type of the intervention (sucralose, stevia, aspartame, saccharine, cyclamate, combined), participant’s health condition (healthy, overweight and obese, diabetic), sex (female, both), type of study (parallel, cross-over), and duration (<12 weeks, ≥12 weeks for total energy intake and <10 weeks, ≥10 weeks for macronutrients intake). Additionally, the subgroup analysis was done based on the type of diet during the intervention (usual diet, low calorie diet) in NNSs vs. water. By performing sensitivity analysis, dependency of the results on the studies was checked. Indeed, to assess the stability of the results, a sensitivity analysis was performed by systematically excluding one study at a time. Evaluation of publication bias was done using Begg’s and Egger’s tests, complemented by a funnel plot depiction. Statistical analyses were carried out in STATA version 17 (Stata Corp, College Station, TX), with statistical significance attributed to *p*-values less than 0.05.

### 2.5 Certainty of evidence

The certainty in evidence was evaluated across trials using the guidelines of the GRADE (Grading of Recommendations Assessment, Development, and Evaluation) working group by two researchers (KR, FM), independently (30). According to the corresponding evaluation criteria, the quality of evidence was divided into high, moderate, low and very low based on risk of bias, inconsistency, indirectness, imprecision, and other considerations such as publication bias, effect size, and potential confounding (31).

## 3 Results

**Figure 1:**
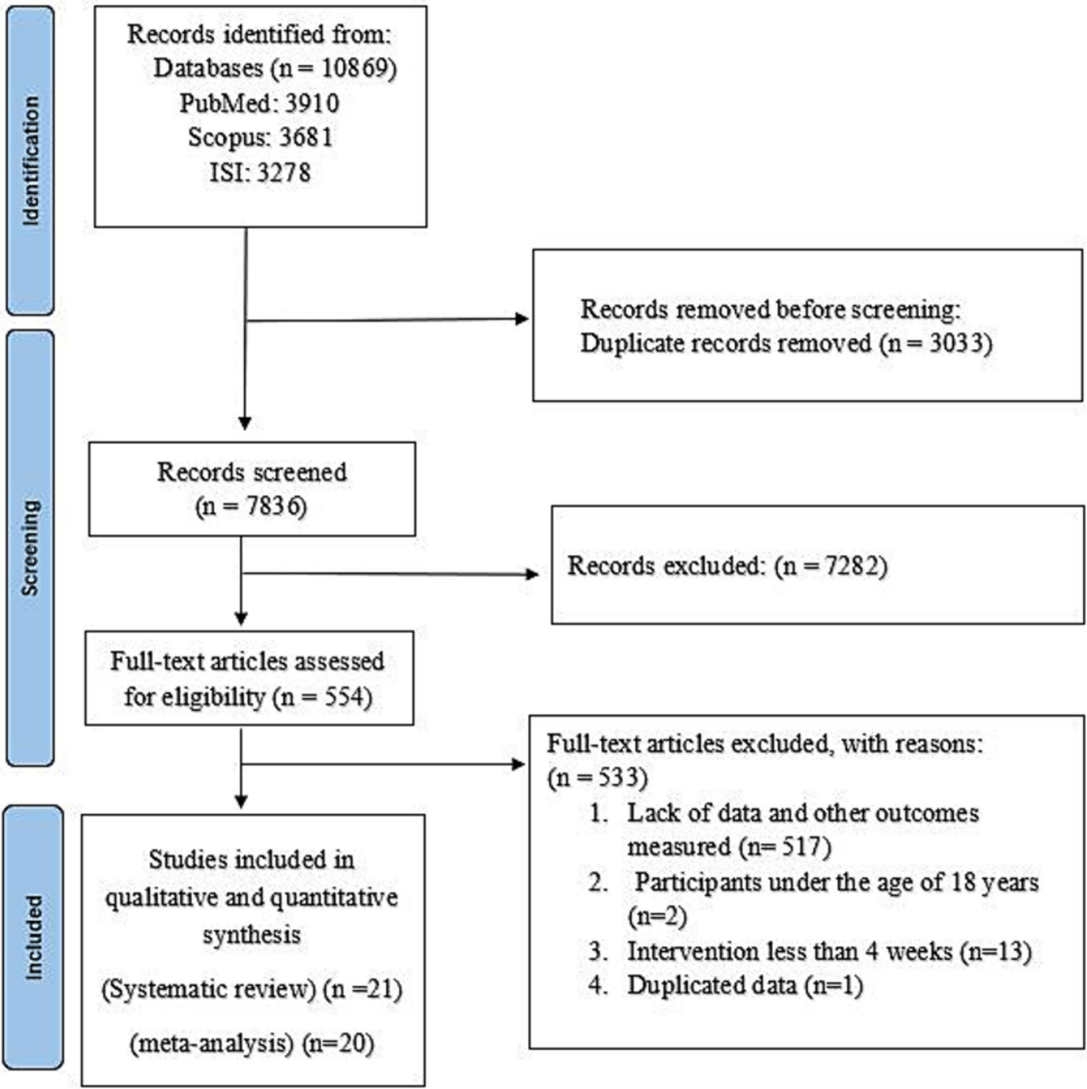
Flow diagram representing the study selection process.

Out of 10,869 references that were found in our primary search, 3,033 duplicate records were excluded. After reviewing the 7,836 remaining articles, 7,282 were discarded as they were deemed irrelevant after examining their titles and abstracts. Finally, after reviewing 554 full-text papers, 533 articles were excluded for the following reasons: 517 studies did not report sufficient or relevant data, 2 studies were conducted on participants under 18 years (32, 33), and 13 studies were assessed the short-term effect of NNSs (less than 4 weeks) (34–46). Two of the included articles had similar datasets (47, 48); therefore, data extraction was done from the most complete one (48). Finally, 21 papers were found to fulfill the inclusion criteria for the systematic review, and 20 papers were appropriate for meta-analysis (16–18, 48–64). The selection process for the study is depicted in Figure 1.

### 3.1 Study characteristics

The characteristics of 21 included RCTs published between 1985 and 2024 are illustrated in Table 1. Eligible studies were conducted in Mexico (49–51, 65), United Kingdom (18, 57, 60, 61), Iran (55, 56), Denmark (48, 53), United States (17, 52, 64), Turkey (16), Switzerland (62), France (54), Canada (59), India (63), and Germany (58). Two of the studies employed a cross-over design (54, 58), while the others had a parallel design (16–18, 48–53, 55–57, 59–65). Fifteen studies were conducted on both genders (17, 18, 48–54, 58, 59, 62–65), and six studies were performed only on female participants (16, 55–57, 60, 61). The characteristics of the participants were as follow: obese or overweight (17, 48, 52–54, 56, 60–62), healthy (16, 18, 49–51, 57, 59, 64, 65), and type 2 diabetes mellitus (55, 58, 63). The age range of the participants was 18 to 65 years, and the duration of the intervention varied between 4 and 52 weeks. Types of artificial sweeteners used include sucralose (16, 49–52, 63), stevia (18, 49, 51, 52, 59), saccharine (16, 52), aspartame (52, 57, 60, 61), cyclamate (58), and combined nonnutritive sweeteners (16, 17, 48, 53–56, 62, 64, 65).

**Table 1:** Characteristics of randomized clinical trials included in the systematic review.^1^

First author (Publication year)	Number of participants and their sex (M/F)	Country	Age (range or mean ± SD)	Design	Duration (weeks)	Intervention type	Intervention dose	Comparison	Participants	Outcomes
Chantelau et al. (1985)	(M/F) Intervention: 10 Control: 10	Germany	25–43	Cross-over	4	Cyclamate	*Ad libitum* within the limitation set up by the WHO (not more than 11 mg/kg body weight/day)	Sucrose	Type 1 (insulin dependent) diabetic patients	Fat intake/ Carbohydrate intake/ Protein intake
Raben et al. (2002)	(M/F) Intervention: 20 Control: 21	Denmark	Intervention: 37.1 ± 9.83 Control: 33.3 ± 9.16	Parallel	10/5	Artificially sweetened drinks and foods	0.57 g/d	Sucrose	Overweight	Energy intake/ Fat intake/ Carbohydrate intake/ Protein intake/ Sugar intake/ Fiber intake
Reid et al. (2007)	(F) Intervention: 66 Control: 67	UK	20–55	Parallel	4	Aspartame	1,000 mL/day	Sucrose	Normal weight	Energy intake/ Fat intake/ Carbohydrate intake/ Protein intake
Reid et al. (2010)	(F) Intervention: 29 Control: 24	UK	Intervention: 32.93 ± 8.84 Control: 34.46 ± 11.03	Parallel	4	Aspartame	1,000 mL/day	Sucrose	overweight	Energy intake/ Fat intake/ Carbohydrate intake/ Protein intake/ Sugar intake
Piernas et al. (2013)	(M/F) Intervention: 84 Control: 85	USA	Intervention: 41.3 ± 103.56 Control: 43.3 ± 97.72	Parallel	26	Diet beverages	Four 340–454 mL/day (12–16 oz)	Water	Overweight/ Obese	Energy intake/ Fat intake/ Carbohydrate intake/ Protein intake/ Sugar intake
Reid et al. (2014)	(F) Intervention: 21 Control: 20	UK	Intervention: 34.6 ± 8.5 Control: 35.1 ± 9.9	Parallel	4	Aspartame	1,000 mL/day	Sucrose	Obese	Energy intake/ Fat intake/ Carbohydrate intake/ Protein intake/ Sugar intake
Campos et al. (2015)	(M/F) Intervention: 14 Control: 13	Switzerland	NR	Parallel	12/6	Artificially sweetened beverages (ASB)	NR	Sugar-sweetened beverage (SSB)	Overweight/ Obesity	Energy intake/ Fat intake/ Carbohydrate intake/ Protein intake/ Sugar intake
Madjd et al. (2015)	(F) Intervention: 32 Control: 30	Iran	Intervention: 37.1 ± 6.8 Control: 32.2 ± 6.9	Parallel	24	Diet beverages	250 mL/day_5 times a week	Water	Overweight/ Obese	Energy intake/ Fat intake/ Carbohydrate intake/ Protein intake/ Fiber intake
Madjd et al. (2016)	(F) Intervention: 40 Control: 41	Iran	Intervention: 35.45 ± 7.45 Control: 34.15 ± 6.99	Parallel	24	Diet beverages	250 mL/day_5 times a week	Water	Type 2 diabetes mellitus	Energy intake/ Fat intake/ Carbohydrate intake/ Protein intake/ Fiber intake
Vázquez-Durán et al. (2016)	(M/F) Intervention: 49 Control: 49	Mexico	Intervention: 21.46 ± 0.31 Control: 22.55 ± 0.51	Parallel	26	Non-calorie sweetened beverages	NR	Without sweetened beverages	Healthy	Energy intake/ Fat intake/ Carbohydrate intake/ Protein intake
Engel et al. (2017)	(M/F) Intervention: 15 Control: 14/16	Denmark	Intervention: 39 ± 7.6 Control: 37.8 ± 8 39 ± 7.3	Parallel	26	Aspartame-sweetened diet cola (NCSD)	NR	Sucrose-sweetened regular cola/ Water	Overweight/ Obesity	Energy intake/ Fat intake/ Carbohydrate intake/ Protein intake
Bonnet et al. (2018)	(M/F) Intervention: 50 Control: 50	France	Intervention: 31 ± 10.3 Control: 31 ± 10.3	Cross-over	12	High intensity sweetened (258 mg aspartame and 26 mg acesulfame K)	660 mL /day (11.16-ounce)	Unsweetened beverage (330 mL carbonated water)	Healthy overweight and non-overweight	Energy intake/ Fat intake/ Carbohydrate intake/ Protein intake/ Sugar intake
Higgins et al. (2019)	(M/F) Intervention: 29/30/28/27 Control: 39	USA	Intervention: 25.8 ± 6.9/29.5 ± 12/27.1 ± 9.6/; 25.9 ± 9 Control: 22.8 ± 9.5	Parallel	12/8	Saccharin/ Aspartame/ RebA/ Sucralose	1.25–1.75 L/d	Sucrose	Overweight/ Obesity	Energy intake
Sánchez-Delgado et al. (2019)	(M/F) Intervention: 13/13 Control: 12	Mexico	Intervention: 22.3 ± 4.4/23.9 ± 5.1 Control: 22.3 ± 3.8	Parallel	6	Sucralose/ Steviol glycosides	0.1 g/day steviol glycosides, 0.048 g/day sucralose	Sucrose	Healthy	Energy intake/ Fat intake/ Carbohydrate intake/ Protein intake
Bueno-Hernández et al. (2020)	(M/F) Intervention: 31/30 Control: 34	Mexico	Intervention: 22.6 ± 2.8/22.9 ± 3.5 Control: 22 ± 3.2	Parallel	10	Sucralose	96 mg/46 mg	Water	Healthy	Energy intake/ Fat intake/ Carbohydrate intake/ Protein intake
Ebbeling et al. (2020)	(M/F) Intervention: 60 Control: 60 /65	USA	Intervention: 26.7 ± 5.7 Control: 25.9 ± 5.1/ 27.9 ± 6	Parallel	52	Artificially sweetened beverage (ASB)	NR (free)	Sugar-sweetened beverage (SSB) / Water	Healthy	Energy intake/Sugar intake
Stamataki et al. (2020)	(M/F) Intervention: 14 Control: 14	UK	Intervention: 25 ± 6 Control: 25 ± 4	Parallel	6/12	Stevia	10 drops daily	Usual diet	Healthy	Energy intake/ Fat intake/ Carbohydrate intake/ Protein intake/ Sugar intake/ Fiber intake
López-Meza et al. (2021)	(M/F) Intervention: 13/13 Control: 13	Mexico	Intervention: 22.23 ± 4.69/23.31 ± 4.8 Control: 21.69 ± 3.58	Parallel	6	Sucralose/ Steviol glycosides	NR	Balanced Deficit Diet (BDD)	Overweight and obesity	Energy intake/ Fat intake/ Carbohydrate intake/ Protein intake
Orku et al. (2023)	(F) Intervention: 11/11/11 Control: 9	Turkey	Intervention: 21.18 ± 1.4/ 21.82 ± 3.16/ 21.64 ± 2.54 Control: 20.11 ± 1.05	Parallel	4	Saccharine/ sucralose/ aspartame and acesulfame-K	140 mg saccharine/ 66 mg sucralose/ 88 mg aspartame and 88 mg acesulfame-K	water	Healthy	Energy intake/ Fat intake/ Carbohydrate intake/ Protein intake
Mohan et al. (2024)	(M/F) Intervention: 91 Control: 88	India	Intervention: 45 ± 6 Control: 45 ± 6	Parallel	12	Sucralose	sucralose-based tabletop sweetener in the pellet (1 pellet = 0.085 g), powder (1 measured spoon = 0.5 g), or liquid form (1 drop = 0.05 mL)	Sucrose	Type 2 diabetes mellitus	Energy intake/ Fat intake/ / Protein intake/Sugar intake
Kwok et al. (2024)	(M/F) Intervention: 31 Control: 32	Canada	Intervention: 30.7 ± 8.4 Control: 31.8 ± 10.3	Parallel	4	Stevia	620 ppm steviol glycosides (equivalent to 75.6 mg steviol)	Sucrose	Healthy	Energy intake/ Fat intake/ Carbohydrate intake/ Protein intake/Fiber intake/Sugar intake

1 NR, not reported; F, female; M, male.

### 3.2 Risk of bias assessment

According to the ROB2 tool, 18 studies were classified as having some concerns (17, 18, 48–52, 54–58, 60–65), two studies were graded as high risk of bias (16, 53), and only one studies had a low risk of bias (59). The quality assessment of the included articles is indicated in Table 2.

**Table 2:** Study quality and risk of bias assessment using ROB 2 tool.

Author	Randomization process	Deviations from the intended interventions (effect of assignment to intervention)	Deviations from the intended interventions (effect adhering to intervention)	Missing outcome data	Measurement of the outcome	Selection of the reported result	Overall
Chantelau et al. (1985)	Some concerns	Low	Some concerns	Low	Low	Low	Some concerns
Raben et al. (2002)	Some concerns	Low	Some concerns	Low	Low	Low	Some concerns
Reid et al. (2007)	Some concerns	Low	Some concerns	Some concerns	Low	Low	Some concerns
Reid et al. (2010)	Some concerns	Low	Low	Some concerns	Low	Low	Some concerns
Piernas et al. (2013)	Some concerns	Low	Low	Low	Low	Low	Some concerns
Reid et al. (2014)	Some concerns	Low	Some concerns	Low	Low	Low	Some concerns
Campos et al. (2015)	Some concerns	Low	Low	Some concerns	Low	Low	Some concerns
Madjd et al. (2015)	Low	Low	Some concerns	Low	Low	Low	Some concerns
Madjd et al. (2016)	Low	Low	Some concerns	Low	Low	Low	Some concerns
Vázquez-Durán et al. (2016)	Some concerns	Low	Some concerns	Low	Low	Low	Some concerns
Engel et al. (2017)	Some concerns	High	Some concerns	Low	Low	Low	High
Bonnet et al. (2018)	Some concerns	Low	Low	Low	Low	Low	Some concerns
Higgins et al. (2019)	Some concerns	Low	Some concerns	Low	Low	Low	Some concerns
Sánchez-Delgado et al. (2019)	Some concerns	Low	Some concerns	Low	Low	Low	Some concerns
Bueno-Hernández et al. (2020)	Some concerns	Some concerns	Low	Some concerns	Low	Low	Some concerns
Ebbeling et al. (2020)	Some concerns	Low	Some concerns	Low	Low	Low	Some concerns
Stamataki et al. (2020)	Some concerns	Low	Some concerns	Low	Low	Low	Some concerns
López-Meza et al. (2021)	Some concerns	Low	Some concerns	Low	Low	Low	Some concerns
Orku et al. (2023)	Some concerns	Low	Some concerns	High	Low	Low	High
Kwok et al. (2024)	Low	Low	Low	Low	Low	Low	Low
Mohan et al. (2024)	Some concerns	Some concerns	Low	Some concerns	Low	Low	Some concerns

### 3.3 Findings from the meta-analysis

#### 3.3.1 The effects of NNSs consumption on total energy intake

**Figure 2:**
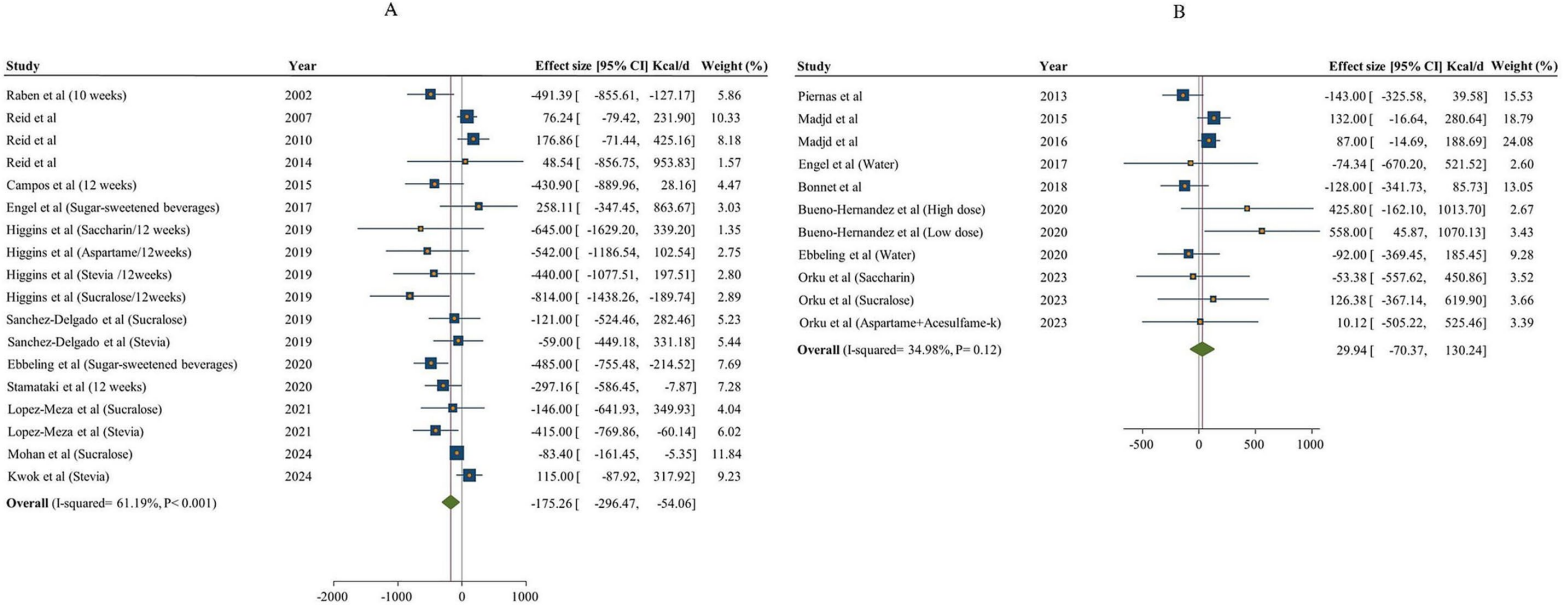
Forest plot expressing the effects of NNSs on energy intake compared to sugar (A) and water (B) intake as control. The analysis was performed using a random effects model.

The extracted data from individual studies are provided Supplementary Table 2. The meta-analysis was performed separately based on the type of control group (sugar and water; Figure 2). In total, 13 articles (18 effect sizes, 944 participants) investigated the effect of NNSs consumption on total energy intake in comparison with sugar (18, 48, 49, 51–53, 57, 59–64), and 8 studies (11 effect sizes, 655 Participants) used water as a control (15–17, 50, 53–56, 64). The results showed a significant decrease in total energy intake [total energy intake change = −175.26 kcal/day, 95% confidence interval (CI): −296.47 to −54.06, I^2^ = 61.19%] after NNS consumption compared to sugar intake. However, there was no significant effect on total energy intake after the NNS intake compared to water (total energy intake change = 29.94 kcal/day, 95% CI: −70.37 to 130.24, I^2^ = 34.98%). The between-study heterogeneity was notable for total energy intake in NNSs intervention vs. both water and sugar comparison (Q statistic _sugar_ = 43.18, Cochrane Q test, *p* < 0.001, I^2^ = 61.19% / Q statistic _water_ = 15.38, Cochrane Q test, *p* = 0.12, I^2^ = 34.98%). Therefore, subgroup analyses were performed to detect potential sources of heterogeneity. In NNSs vs. sugar, the result indicated that among different types of NNS as intervention, only combined NNSs significantly decreased total energy intake (*p* < 0.001). In studies that included both male and female participants together, NNSs vs. sugar demonstrated a significant reduction in total energy intake (total energy intake change: −248.56 kcal/day; 95% CI: −384.69 to −112.44, I^2^ = 57%). However, this effect was not statistically significant in studies that focused exclusively on female participants (*p* = 0.12). In NNSs vs. sugar, the result showed a significant decrease in total energy intake among participants with diabetes [Weighted mean difference (WMD): −83.40 kcal/day (95% CI: −161.45 to −5.35, *p* = 0.04]. Both short-term (shorter than 12 weeks) and long-term intervention (12 weeks and more) with NNSs vs. sugar revealed remarkable reduction in total energy intake (*p* < 0.05). In NNSs vs. water, the subgroup analysis indicated that only sucralose as NNSs significantly increased total energy intake (WMD = 358.71 kcal/day, 95% CI: 54.59 to 662.83, I^2^ = 0%), while the other NNSs had no significant effect. Moreover, when NNSs was compared with water in the context of a low-calorie diet, a higher total energy intake was observed in the NNS intake group (*p* = 0.02). The overall results based on different comparisons are shown in Table 3.

**Table 3:** Meta-analysis showing the effect of non-nutritive sweeteners consumption on energy intake based on several subgroups (all analyses were conducted using random effects model).

‌			Meta-analysis		Heterogeneity			
	Study group	No. of studies/effect sizes	WMD^1^ (95%CI)	*P* effect	*Q* statistic	*P* within group	*I^2^* (%)	*P* between group
NNSs vs. sugar								
Duration	<12 weeks	10/15	−143.82 (−283.86, −3.78)	0.04	28.99	0.01	51.71	0.09
	≥12 weeks	7/10	−348.77 (−540.06, −157.47)	< 0.001	23.70	< 0.001	62.02	
Type of intervention	Sucralose	4/4	−179.98 (−408.51, 48.55)	0.12	5.24	0.16	42.70	< 0.001
	Combined	3/3	−476.96 (−673.26, −280.67)	< 0.001	0.05	0.98	0	
	Stevia	5/5	−175.80 (−418.02, 66.04)	0.15	10.26	0.04	61	
	Saccharine	1/1	−645 (−1629.20, 339.20)	0.20	0	–	–	
	Aspartame	5/5	83.39 (−66.26, 233.04)	0.27	4.49	0.34	10.98	
Gender	Female	3/3	103.46 (−27.05, 233.97)	0.12	0.47	0.79	0	< 0.001
	Both	10/15	−248.56 (−384.69, −112.44)	< 0.001	32.56	< 0.001	57	
Characteristic of participants	‌ Healthy	6/8	−151.03 (−330.63, 28.56)	0.10	21.74	< 0.001	67.80	0.33
	Obese and overweight	6/9	−292 (−578, −6)	0.05	20.82	0.01	61.57	
	Diabetes	1/1	−83.40 (−161.45, −5.35)	0.04	0	–	–	
overall		13/18	−175.26 (−296.47, −54.06)	< 0.001	43.18	< 0.001	61.19	
NNSs vs. water								
Duration	<12 weeks	2/5	200.30 (−32.74, 433.34)	0.09	4.02	0.40	0.52	0.12
	≥12 weeks	6/6	−3.62 (−112.03, 104.79)	0.95	9.42	0.09	46.93	
Type of intervention	Sucralose	2/3	358.71 (54.59, 662.83)	0.02	1.48	0.47	0	0.17
	Combined	6/6	−0.50 (−107.48, 106.48)	0.99	9.31	0.09	46.31	
	Saccharine	1/1	−53.38 (−557.62, 450.86)	0.84	0	–	–	
	Aspartame	1/1	−74.34 (−670.20, 521.52)	0.81	0	–	–	
Gender	Female	3/5	95.82 (15.18, 176.46)	0.02	0.71	0.95	0	0.29
	Both	5/6	−11.79 (−194.14, 170.55)	0.90	9.43	0.09	46.98	
Characteristic of participants	‌ Healthy	3/6	107.55 (−105.37, 320.94)	0.32	6.53	0.25	23.38	0.41
	Obese and overweight	4/4	−37.41 (−204.59, 129.76)	0.66	6.76	0.08	55.60	
	Diabetes	1/1	87 (−14.68, 188.68)	0.09	0	–	–	
Type of study	Parallel	7/10	53.33 (−49.03, 155.70)	0.31	12.69	0.17	29.07	0.13
	Crossover	1/1	−128 (−341.73, 85.73)	0.24	0	–	–	
Type of diet during intervention	Usual diet	6/8	−28.20 (−160.42, 104)	0.68	10.07	0.26	20.53	0.10
	low calorie diet	2/2	101.34 (17.42, 185.27)	0.02	0.24	0.62	0	
overall		8/11	29.94 (−70.37, 130.24)	0.56	15.38	0.12	34.98	

^1WMD: Weighted mean difference.^

#### 3.3.2 The effects of NNSs consumption on carbohydrate intake

**Figure 3:**
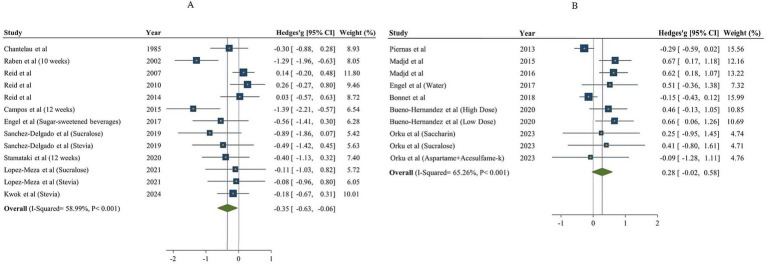
Forest plot indicating the effects of NNSs on carbohydrate intake compared to sugar (A) and water (B) intake as a control. The analysis was performed using a random effects model.

A total of 11 articles with 13 effect sizes and 502 participants assessed the effects of NNSs consumption on carbohydrate intake (18, 48, 49, 51, 53, 57–62). The comparison of NNSs with sugar showed a significant reduction in carbohydrate intake [Hedges’ g = −0.35, 95% CI: −0.63 to −0.06, I^2^ = 58.99%]. In contrast, the comparison of NNSs with water, based on 7 articles (10 effect sizes, 530 participants) (16, 17, 50, 53–56), indicated no significant effect on carbohydrate intake [Hedges’ g = 0.28, 95% CI: −0.02 to 0.58, I^2^ = 65.26%, Figure 3]. High between-study heterogeneity was observed for the effect of NNSs intake on carbohydrate intake compared with both sugar and water (Q statistic _sugar_ = 29.26, Cochrane Q test, *p* < 0.001, I^2^ = 58.99%/ Q statistic _water_ = 25.91, Cochrane Q test, *p* < 0.001, I^2^ = 65.26%). For NNSs vs. sugar, subgroup analysis demonstrated that consuming NNSs for over 10 weeks led to a notable decrease in carbohydrate intake (Hedges’ g = −0.92, 95% CI: −1.42 to −0.43, I^2^ = 41.35%). Moreover, this significant effect was only seen after consumption of combined NNSs (Hedges’ g = −1.33, 95% CI: −1.85 to −0.82, I^2^ = 0%), not with other types of NNSs. A significant reduction was also seen in parallel design studies and in studies including both genders. In NNSs vs. water, only sucralose intake had significant effect on carbohydrate intake (Hedges’ g = 0.54, 95% CI: 0.14 to 0.93, I^2^ = 0%). In NNSs vs. water, the result showed a significant increase in carbohydrate intake among female participants (*p* < 0.001). The significant effect of NNSs in comparison with water was also seen in healthy and diabetic participants (*p* = 0.01). Prescribing a low-calorie diet during the intervention significantly affected carbohydrate intake. The result of meta-analysis based on both comparison (sugar and water) is indicated in Table 4.

**Table 4:** Meta-analysis showing the effect of non-nutritive sweeteners consumption on carbohydrate intake based on several subgroups (all analyses were conducted using random effects model).

‌			Meta-analysis		Heterogeneity			
	Study group	No. of studies/ effect sizes	Hedges’ g^1^ (95%CI)	*P* effect	*Q* statistic	*P* within group	*I^2^* (%)	*P* between group
NNSs vs. sugar								
Duration	<10 weeks	10/12	−0.15 (−0.38, 0.08)	0.19	16.94	0.11	35.08	0.01
	≥10 weeks	4/4	−0.92 (−1.42, −0.43)	<0.001	5.12	0.16	41.35	
Type of intervention	Sucralose	2/2	−0.49 (−1.26, 0.28)	0.21	1.33	0.25	24.55	<0.001
	Combined	2/2	−1.33 (−1.85, −0.82)	<0.001	0.03	0.86	0	
	Stevia	4/4	−0.26 (−0.60, 0.09)	0.14	0.63	0.89	0	
	cyclamate	1/1	−0.30 (−0.88, 0.28)	0.32	0	–	–	
	Aspartame	4/4	0.09 (−0.16, 0.33)	0.49	2.73	0.43	0	
Gender	Female	3/3	0.15 (−0.11, 0.40)	0.27	0.34	0.85	0	<0.001
	Both	8/10	−0.55 (−0.85, −0.25)	<0.001	14.39	0.11	37.47	
Characteristic of participants	‌ Healthy	5/7	−0.11 (−0.33, 0.12)	0.36	5.92	0.43	0	0.42
	Obese and overweight	5/5	−0.56 (−1.25, 0.13)	0.11	20.56	<0.001	80.54	
	Diabetes	1/1	−0.30 (−0.88, 0.28)	0.32	0	–	–	
Type of study	parallel	10/12	−0.36(−0.67, −0.04)	0.03	29.20	<0.001	62.33	0.85
	Crossover	1/1	−0.30 (−0.88, 0.28)	0.32	0	–	–	
overall		11/13	−0.35 (−0.63, −0.06)	0.02	29.26	<0.001	58.99	
NNSs vs. water								
Duration	<10 weeks	1/3	0.19 (−0.50, 0.88)	0.59	0.34	0.84	0	0.77
	≥10 weeks	6/7	0.30 (−0.05, 0.68)	0.09	25.52	<0.001	76.49	
Type of intervention	Sucralose	2/3	0.54 (0.14, 0.93)	0.01	0.27	0.87	0	0.59
	Combined	5/5	0.15 (−0.26, 0.57)	0.48	19.09	0.001	79.04	
	Saccharine	1/1	0.24 (−0.94, 1.44)	0.68	0	–	–	
	Aspartame	1/1	0.50 (−0.36, 1.37)	0.25	0	–	–	
Gender	Female	3/5	0.55 (0.25, 0.85)	<0.001	1.71	0.78	0	0.07
	Both	4/5	0.13 (−0.23, 0.50)	0.47	12.76	0.01	68.66	
Characteristic of participants	‌ Healthy	2/5	0.45 (0.10, 0.81)	0.01	1.36	0.85	0	0.21
	Obese and overweight	4/4	0.09 (−0.32, 0.52)	0.65	12.31	0.006	75.63	
	Diabetes	1/1	0.62 (0.18, 1.06)	0.01	0	–	–	
Type of study	parallel	6/9	0.36 (0.02, 0.70)	0.04	20.55	0.008	61.06	0.02
	Crossover	1/1	−0.15 (−0.42, 0.12)	0.27	0	–	–	
Type of diet during intervention	Usual diet	5/8	0.12 (−0.17, 0.41)	0.42	13.51	0.06	48.19	0.02
	low calorie diet	2/2	0.64 (0.31, 0.97)	<0.001	0.02	0.88	0	
overall		7/10	0.28 (−0.02, 0.58)	0.07	25.91	<0.001	65.26	

^1Hedges’ g: Bias corrected standardized mean difference.^

#### 3.3.3 The effects of NNSs consumption on protein intake

**Figure 4:**
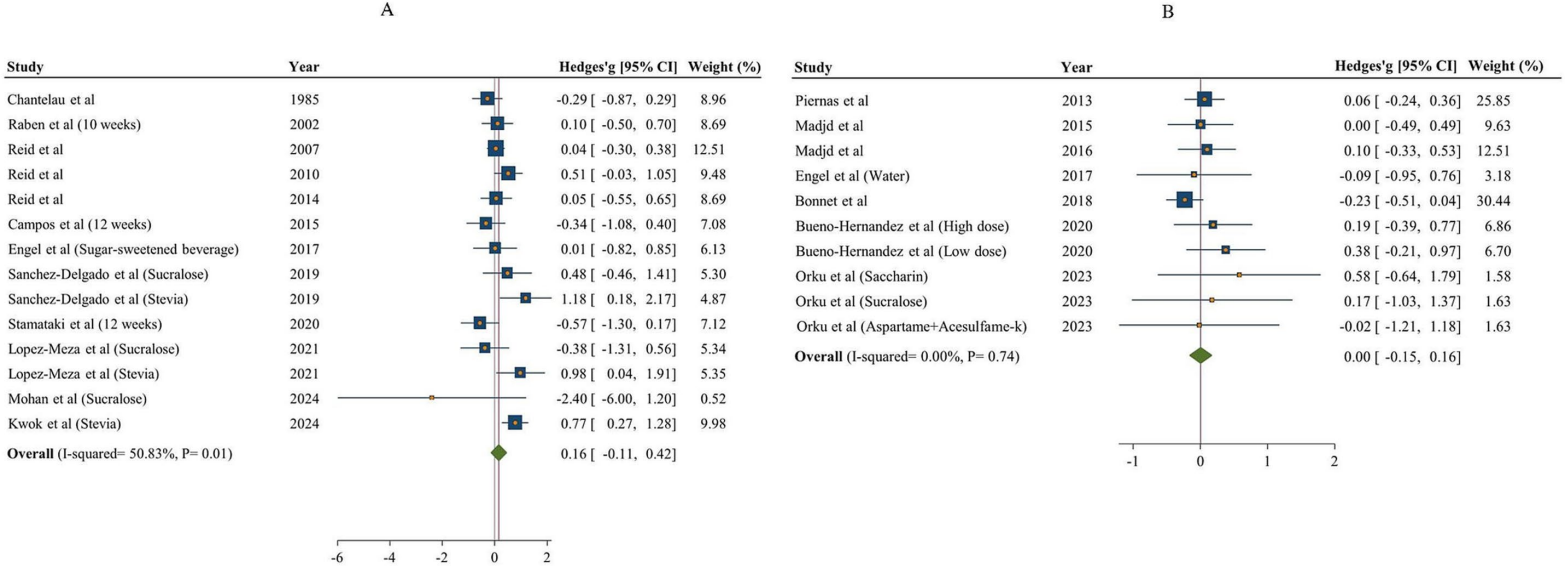
Forest plot showing the effects of NNSs on protein intake compared to sugar (A) and water (B) intake as a control. The analysis was performed using a random effects model.

After analyzing the results of 12 studies with 14 effect sizes and 681 participants for NNSs vs. sugar (18, 48, 49, 51, 53, 57–63), and 7 studies (10 effect sizes, 530 participants) for NNSs vs. water (16, 17, 50, 53–56), no significant effect was found on protein intake following NNSs consumption in comparison with both sugar and water intake [Hedges’ g _sugar_ = 0.16, 95% CI: −0.11 to 0.42, I^2^ = 50.83%] /[Hedges’ g _water_ = 0.00, 95% CI: −0.15 to 0.16, I^2^ = 0%, Figure 4]. Between-study heterogeneity was observed only for NNSs vs. sugar intake (Q statistic =26.44, Cochrane Q test, *p* = 0.01, I^2^ = 50.83%). Subgroup analyses were conducted to find probable source of heterogeneity, but the effect was not significant in any subgroups (Table 5).

**Table 5:** Meta-analysis showing the effect of non-nutritive sweeteners consumption on protein intake based on several subgroups (all analyses were conducted using random effects model).

‌			Meta-analysis		Heterogeneity			
	Study group	No. of Studies/ effect sizes	Hedges’ g^1^ (95%CI)	*P* effect	*Q* statistic	*P* within group	*I^2^* (%)	*P* between group
NNSs vs. sugar								
Duration	<10 weeks	10/12	0.23 (−0.03, 0.49)	0.08	21.38	0.03	48.55	0.05
	≥10 weeks	5/5	−0.20 (−0.55, 0.16)	0.28	3.73	0.44	0	
Type of intervention	Sucralose	3/3	−0.11 (−1.06, 0.84)	0.82	3.32	0.19	39.77	0.43
	Combined	2/2	−0.08 (−0.54, 0.39)	0.75	0.83	0.36	0	
	Stevia	4/4	0.56 (−0.20, 1.32)	0.15	11.93	0.01	74.85	
	cyclamate	1/1	−0.29 (−0.87, 0.29)	0.33	0	–	–	
	Aspartame	4/4	0.14 (−0.11, 0.38)	0.28	2.35	0.50	0	
Gender	Female	3/3	0.16 (−0.12, 0.44)	0.28	2.26	0.32	11.33	0.95
	Both	9/11	0.14 (−0.24, 0.52)	0.46	24.18	0.01	58.64	
Characteristic of participants	‌ Healthy	5/7	0.32 (−0.13, 0.78)	0.16	17.98	0.01	66.62	0.46
	Obese and overweight	5/5	0.13 (−0.16, 0.41)	0.38	3.64	0.46	0	
	Diabetes	2/2	−0.56 (−1.97, 0.84)	0.43	1.29	0.26	22.52	
Type of study	parallel	11/13	0.20 (−0.08, 0.48)	0.16	24.03	0.02	50.07	0.14
	Crossover	1/1	−0.29 (−0.87, 0.29)	0.33	0	–	–	
overall		12/14	0.16 (−0.11, 0.42)	0.25	26.44	0.01	50.83	
NNSs vs. water								
Duration	<10 weeks	1/3	0.24 (−0.45, 0.93)	0.5	0.49	0.78	0	0.49
	≥10 weeks	6/7	−0.009 (−0.16, 0.14)	0.91	5.06	0.53	0	
Type of intervention	Sucralose	2/3	0.26 (−0.12, 0.66)	0.18	0.22	0.89	0	0.37
	Combined	5/5	−0.05 (−0.22, 0.11)	0.53	2.68	0.61	0	
	Saccharine	1/1	0.57 (−0.63, 1.79)	0.35	0	–	–	
	Aspartame	1/1	−0.09 (−0.95, 0.76)	0.83	0	–	–	
Gender	Female	3/5	0.08 (−0.20, 0.38)	0.56	0.80	0.93	0	0.59
	Both	4/5	−0.01 (−0.21,0.19)	0.92	4.78	0.31	16.38	
Characteristic of participants	‌ Healthy	2/5	0.27 (−0.08, 0.62)	0.14	0.70	0.95	0	0.2
	Obese and overweight	4/4	−0.08 (−0.26, 0.09)	0.37	2.10	0.55	0	
	Diabetes	1/1	0.09 (−0.33, 0.52)	0.66	0	–	–	
Type of study	parallel	6/9	0.10 (−0.07, 0.29)	0.25	2	0.98	0	0.04
	Crossover	1/1	−0.23 (−0.51, 0.04)	0.10	0	–	–	
Type of diet during intervention	Usual diet	5/8	−0.01 (−0.18, 0.16)	0.90	5.81	0.56	0	0.72
	low calorie diet	2/2	0.05 (−0.26, 0.37)	0.74	0.08	0.77	0	
overall		7/10	0.00 (−0.15, 0.16)	0.97	6.02	0.74	0	

^1Hedges’ g: Bias corrected standardized mean difference.^

#### 3.3.4 The effect of NNSs consumption on fat intake

**Figure 5:**
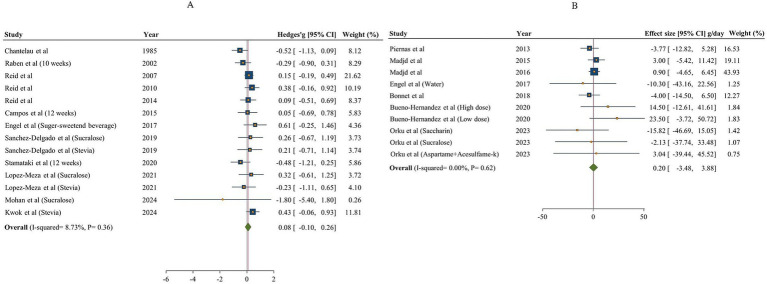
Forest plot showing the effects of NNSs on fat intake compared to sugar (A) and water (B) intake as a control. The analysis was performed using a random effects model.

The overall result of NNSs consumption on fat intake, based on comparisons with both sugar and water is shown in Figure 5. Totally, 12 articles (14 effect sizes) examined the effects of NNSs intake on fat intake in comparison with sugar (15, 18, 48, 49, 53, 57–63). No significant effect on fat intake was found following NNSs consumption in comparison with sugar [Hedges’ g _sugar_ = 0.08, 95% CI: −0.10 to 0.26, I^2^ = 8.73%]. Similarly, no significant effect was observed for NNSs vs. water [fat intake change _water_ = 0.20 g/day, 95% CI: −3.48 to 3.88, I^2^ = 0%]. The between-study heterogeneity was not notable for fat intake in comparisons of NNSs with both sugar and water.

#### 3.3.5 The effects of NNSs consumption on sugar and fiber intake

**Figure 6:**
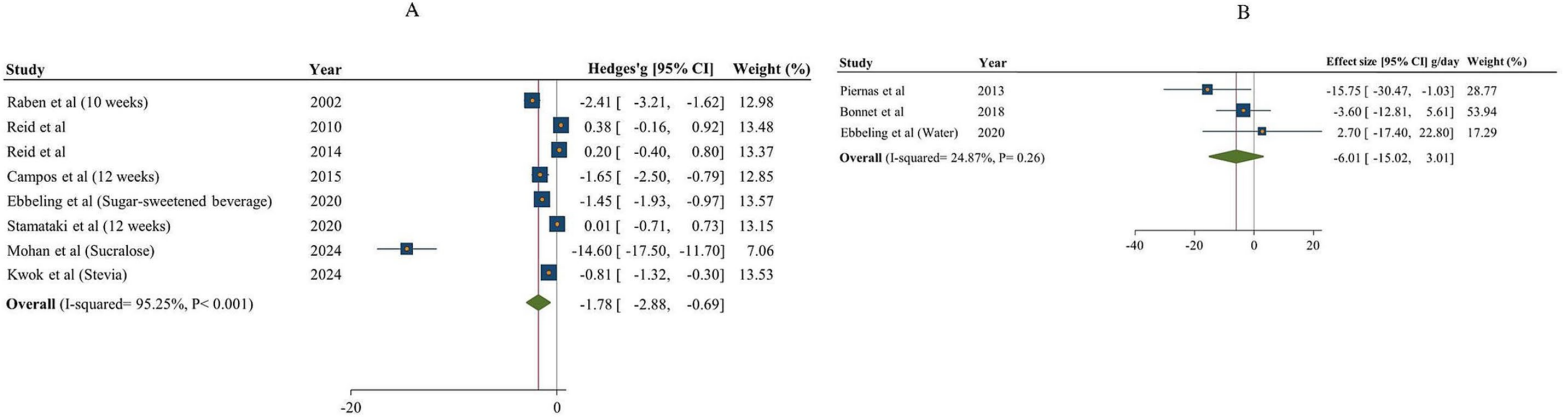
Forest plot illustrating the effects of NNSs on sugar intake compared to sugar (A) and water (B) intake as a control. The analysis was performed using a random effects model.

The overall findings on the impact of non-nutritive sweeteners (NNSs) on sugar intake, comparing both sugar and water, are illustrated in Figure 6. A total of 8 studies (8 effect sizes and 558 participants) investigated the effects of NNSs on sugar intake compared to sugar (18, 48, 59–64), and 3 studies (3 effect sizes and 339 participants) assessed the effects of NNSs on sugar intake compared to water (17, 54, 64). The results showed a significant reduction in sugar intake when NNSs were consumed instead of sugar (Hedges’ g = −1.78, 95% CI: −2.88 to −0.69, I^2^ = 95.25%). Conversely, there was no significant effect when comparing NNSs to water (sugar intake change = −6.01 g/day, 95% CI: −15.02 to 3.01, I^2^ = 24.87%). Additionally, there was notable between-study heterogeneity in sugar intake for NNSs compared to sugar (Q statistic =147.29, Cochrane Q test, *p* < 0.001, I^2^ = 95.25%). Type of intervention, duration, characteristics of population, and gender were considered as potential sources of heterogeneity (Table 6).

**Table 6:** Meta-analysis showing the effect of non-nutritive sweeteners consumption on sugar intake based on several subgroups (all analyses were conducted using random effects model).

‌			Meta-analysis		Heterogeneity			
	Study group	No. of effect sizes	Hedges’ g^1^ (95%CI)	*P* effect	*Q* statistic	*P* within group	*I^2^* (%)	*P* between group
NNSs vs. sugar								
Duration	<10 weeks	6	−0.12 (−0.75, 0.51)	0.71	30.88	<0.001	83.81	<0.001
	≥10 weeks	5	−3.25 (−5.08, −1.43)	<0.001	100.38	<0.001	96.02	
Type of intervention	Combined	3	−1.78 (−2.36, −1.20)	<0.001	4.13	0.13	51.53	<0.001
	Stevia	3	−4.47 (−7.90, −1.05)	0.01	91.75	<0.001	97.82	
	Aspartame	2	0.30 (−0.10, 0.70)	0.14	0.20	0.66	0	
Gender	Female	2	0.30 (−0.10, 0.70)	0.14	0.20	0.66	0	<0.001
	Both	6	−2.62 (−4, −1.25)	<0.001	105.50	<0.001	95.26	
Characteristic of participants	‌ Healthy	3	−0.79 (−1.55, −0.02)	0.04	11.15	<0.001	82.06	<0.001
	Obese and overweight	4	−0.84 (−2.15, 0.47)	0.21	44.52	<0.001	93.26	
	Diabetes	1	−14.60 (−17.50, −11.70)	<0.001	0	–	–	
overall		8	−1.78 (−2.88, −0.69)	<0.001	147.29	<0.001	95.25	–
		No. of effect sizes	WMD^2^ (95%CI)	*P* effect	*Q* statistic	*P* within group	*I*^2^ (%)	*P* between group
NNSs vs. water								
overall		3	−6.01 (−15.02, 3.01)	0.19	2.66	0.26	24.87	–

^1Hedges’ g: Bias corrected standardized mean difference.2WMD: Weighted mean difference.^

Five articles (275 participants) provided information on the effects of NNSs on fiber intake (18, 48, 55, 56, 59). The result did not report any significant difference on fiber intake after intervention with NNSs compared to either vs. sugar or water [Hedges’ g _sugar_ = 0.05, 95% CI: −0.28 to 0.39, I^2^ = 0%/ fiber intake change _water_ = −0.01 g/day, 95% CI: −0.27 to 0.26, I^2^ = 0%]. Heterogeneity sources were not found on fiber intake.

### 3.4 Publication bias and sensitivity analysis

Sensitivity analysis revealed that only the overall effect of NNSs vs. sugar on carbohydrate intake changed to non-significant effect after removing the following studies: Raben et al. (48) (Hedges’ g = −0.23; 95% CI: −0.48, 0.01), and Campos et al. (62) (Hedges’ g = −0.26; 95% CI: −0.52, 0.001). No other modification in overall effects was observed after removing each study or studies with a high risk of bias.

Although slight asymmetries were observed in meta-analyses no publication bias was confirmed using asymmetry tests (Begg’s test and Egger’s test, Supplementary Figures 1–11): total energy intake (Begg’s test _NNS vs. sugar_, *p* = 0.32/ Begg’s test _NNS vs. water_, *p* = 0.64; Egger’s test _NNS vs. sugar_, *p* = 0.053/ Egger’s test _NNS vs. water_, *p* = 0.44), Carbohydrate intake (Begg’s test _NNS vs. sugar_, *p* = 0.07/ Begg’s test _NNS vs. water_, *p* = 0.72; Egger’s test _NNS vs. sugar_, *p* = 0.03/ Egger’s test _NNS vs. water_, *p* = 0.34), Fat intake (Begg’s test _NNS vs. sugar_, *p* = 0.66/ Begg’s test _NNS vs. water_, *p* = 1; Egger’s test _NNS vs. sugar_, *p* = 0.35/ Egger’s test _NNS vs. water_, *p* = 0. 86), Protein intake (Begg’s test _NNS vs. sugar_, *p* = 1/ Begg’s test _NNS vs. water,_ *p* = 0.37; Egger’s test _NNS vs. sugar_, *p* = 0.48 / Egger’s test _NNS vs. water_, *p* = 0.19), Sugar intake (Begg’s test _NNS vs. sugar_, *p* = 0.26/Begg’s test _NNS vs. water_, *p* = 1; Egger’s test _NNS vs. sugar_, *p* = 0.00 /Egger’s test _NNS vs. water_, *p* = 0.84). Fiber intake (Begg’s test *p* = 0.80 / Egger’s test *p* = 0.73). We utilized the trim and fill method to assess if the publication bias affected the summary effect for carbohydrate and sugar intake, and found that no studies were trimmed. The overall effects remained unchanged, suggesting the absence of publication bias.

### 3.5 Grading the evidence

The level of confidence after using the GRADE protocol is reported in Table 7. The GRADE assessment for total energy intake vs. sugar was considered as high quality. The level of confidence for carbohydrate intake vs. sugar was evaluated as being moderate due serious limitations in risk of bias and imprecision. However, the evidence relating to other outcomes identified as low due to serious limitations in risk of bias and very serious issues of imprecision.

**Table 7:** Grade profile of non-nutritive sweeteners on energy and nutrient intake.

Certainty assessment						
Outcomes	Risk of bias	Inconsistency	Indirectness	Imprecision	Publication bias	Certainty
Total enery intake vs. sugar	serious^a^	not serious^b^	not serious^c^	Serious^d^	not serious	⨁⨁⨁⨁ High
Total energy intake vs. water	serious^a^	not serious	not serious^c^	very serious^e^	not serious	⨁⨁◯◯ low
Carbohydrate intake vs. sugar	serious^a^	not serious^b^	not serious^c^	Serious^d^	not serious	⨁⨁⨁◯ Moderate
Carbohydrate intake vs. water	serious^a^	not serious	not serious^c^	very serious^e^	not serious	⨁⨁◯◯ low
fat intake vs. sugar	serious^a^	not serious	not serious^c^	very serious^e^	not serious	⨁⨁◯◯ low
fat intake vs. water	serious^a^	not serious	not serious^c^	very serious^e^	not serious	⨁⨁◯◯ low
Protein intake vs. sugar	serious^a^	not serious	not serious^c^	very serious^e^	not serious	⨁⨁◯◯ low
protein intake vs. water	serious^a^	not serious	not serious^c^	very serious^e^	not serious	⨁⨁◯◯ low
Fiber intake	serious^a^	not serious	not serious^c^	very serious^e^	not serious	⨁⨁◯◯ low
Sugar intake	serious^a^	not serious^b^	not serious^c^	Serious^d^	not serious	⨁⨁◯◯ low

a More than 20% of RCTs for this outcome had some concerns of bias for at least one component of ROB2 tool.

b The I2 value was > 50%, however, the high heterogeneity was explained in the subgroup analyses.

c Some trials used a combined intervention of two or more types of non-nutritive sweeteners; however, subgroup analysis was conducted based on type of intervention.

d The optimal information size (OIS) exceeds the sample size of the trials. However, there is no evidence of publication bias.

e The confidence interval does not show statistical significance. Moreover, the optimal information size (OIS) exceeds the sample size of the trials. However, there is no evidence of publication bias.

## 4 Discussion

This systematic review and meta-analysis were done to observe the effects of NNSs consumption in comparison with sugar and water on long-term total energy, fat, carbohydrate, protein, fiber and sugar intake among adults. Our results support the reduction effect of NNSs consumption on total energy, carbohydrates and sugar intake when NSSs were compared with sugar.

There are several meta-analyses regarding the effects of NNSs on energy intake. In a meta-analysis by Santos et al. (66), it was reported that aspartame consumption had no significant effect on energy intake compared to both sucrose or control. Also, the findings from the subgroup analysis in our study showed that aspartame consumption was not associated with a significant alteration in energy intake compared to sugar or water as a control.

In another meta-analysis of 25 RCTs performed by Montez et al. (67) on adults and children for periods longer than 7 days, a notable decrease in daily energy consumption was shown in individuals using NNSs. Also, in agreement with our result, in the investigation conducted by Rogers et al. (19), encompassing both parallel and cross-over studies with a minimum duration of 1 week, a significant reduction in body weight and energy intake was indicated after low-calorie sweeteners (LCS) consumption vs. sugar. However, this significant effect was not observed in the consumption of NNSs compared to water.

Toews et al. (20) conducted a meta-analysis of ‌ four randomized controlled trials (RCTs) consisted a healthy population of both children and adults. The results demonstrated a statistically significant reduction in mean daily caloric intake by approximately 250 kcal in individuals ingesting non-nutritive sweeteners (NNSs) as opposed to those ingesting sucrose. The results of this study are consistent with our study, with the difference that our study was conducted only on the adult population and considering a larger number of articles compared to the study by Toews et al. Existing meta-analyses rarely reached definitive conclusions and mostly examined energy as a secondary factor. In the present meta-analysis, we tried to find all RCT articles related to artificial sweeteners through a comprehensive search. The full text of all related articles was assessed to get the useful results of our opinion. Also, in this study, we examined the effects of NNSs not only on total energy intake but also on the intake of macronutrients, fiber, and sugar. NNSs may have physiological function, affecting nutrition and metabolism in different ways (68). The mechanism of NNSs on energy metabolism is not completely clear, and there are differing opinions on the effect of non-nutritive sweeteners on the energy. The published findings showed that an NNSs pre-load results in energy compensation for the day without leading to overcompensation (38).

The results of current analysis showed that the consumption of non-nutritive sweeteners compared to sugar can lead to a decrease in total energy intake. Supporting our findings, Mohan et al. (63) discovered that consuming sucralose instead of sugar can lead to a reduction in energy intake after a 12-week intervention period. However, the results of this study did not observe any significant effect of sucralose on HbA1c (63). Madjd et al.’s study suggested that substituting diet beverages (DBs) with water after the main meal in obese women with type 2 diabetes may result in greater weight loss during a weight reduction program (55). A network meta-analysis of 36 acute single exposure studies found that NNS beverages can be a suitable alternative to sugar-sweetened beverages (SSBs) in the short-term period after meals (69). A meta-analysis of 29 randomized controlled trials (RCTs), involving 741 participants, investigated the glycemic effects of four non-nutritive sweeteners (NNSs) including saccharin, aspartame, sucralose, and stevia (70), resulting that consuming NNSs did not alter blood glucose levels (70).

The present meta-analysis has certain limitations that should be mentioned. First, according to the Cochrane risk-of-bias tool for randomized trials (RoB2), most of our studies had some concerns, indicating a need for high-quality studies. Second, substantial heterogeneity was observed among studies evaluating the effect of NNS on sugar intake, total energy and carbohydrate intake vs. sugar. Our study also had strengths worth mentioning. We performed a robust and comprehensive search. Also, we examined all types NNSs and compared them in different subgroups. The GRADE assessment was done to better evaluate the quality of the entered articles. Since most evidence suggests that NNSs have no short-term effects on energy intake, we focused on studies examining long-term effects (greater than 4 weeks). Additionally, we examined the effect of NNS consumption on individual macronutrient intake, not just energy intake. In conclusion, the results of the current study suggested that NNSs consumption may be effective in reducing total energy, carbohydrates, and sugar intake vs. sugar. High quality randomized controlled clinical trials are essential to validate our results.

## Data Availability

Publicly available datasets were analyzed in this study. This data can be found here: Information about the data and analysis performed in the present study is available from the corresponding author upon reasonable request.
