# Supplementary material for: The effects of non-nutritive sweeteners on energy and macronutrients intake in adults: a grade-assessed systematic review and meta-analyses of randomized controlled trials
Source: Front Nutr. 2024 Nov 13;11:1475962. doi: 10.3389/fnut.2024.1475962 (PMC11598426; doi:10.3389/fnut.2024.1475962)
Supplement: Supplementary file 1 [file Table_1.DOCX]

**SUPPORTING INFORMATION**

**Legends to supplementary Tables**

**Supplemental Table 1. The strategy used to search for different databases**

| **PubMed**  ("Non-Nutritive Sweeteners"[Mesh] OR "Non-Nutritive Sweeteners"[tiab] OR "Non Nutritive Sweeteners"[tiab] OR “Aspartame"[Mesh] “Aspartame"[tiab] OR "Stevia"[Mesh] OR "Stevia"[tiab] OR "Saccharin"[Mesh] OR "Saccharin"[tiab] OR "acetosulfame" [Supplementary Concept] OR acesulfame K[tiab] OR “acesulfame potassium”[tiab] OR “Ace-K”[tiab] OR “AceK”[tiab] OR "Sorbitol"[Mesh] OR "Sorbitol"[tiab] OR "trichlorosucrose" [Supplementary Concept] OR sucralose[tiab] OR “artificial sweeteners “[tiab] OR "Carbonated Beverages"[Mesh] OR "Carbonated Beverages"[tiab] OR “diet soda”[tiab] OR “Sugary Soda”[tiab] OR “Sugar Sweetened Soda”[tiab] OR “Artificially Sweetened Soda”[tiab] OR “non-caloric sweeteners”[tiab] OR “non caloric sweeteners”[tiab] OR Advantame[tiab] OR Neotame[tiab] OR "neotame" [Supplementary Concept] OR “zero-calorie sweetener”[tiab] OR NutraSweet [tiab] OR Splenda [tiab] OR "Cyclamates"[Mesh] OR "Cyclamates"[tiab] OR cyclamic acid [tiab] OR "acetosulfame"[tiab] OR “high-intensity sweetener” [tiab] OR “Steviol glycosides”[tiab] OR "rebaudioside A" [Supplementary Concept] OR "rebaudioside A" [tiab] OR “reb a”[tiab] OR "stevioside" [Supplementary Concept] OR "stevioside" [tiab] OR "rebaudioside D" [Supplementary Concept] OR "rebaudioside D" [tiab] OR newtame [tiab] OR "sugar twin"[tiab] OR nutrasweet[tiab] OR “necta sweet”[tiab] OR “sweet’n low”[tiab] OR “Luo Han Guo”[tiab] OR “monk fruit”[tiab] OR “Siraitia grosvenorii Swingle fruit extract”[tiab] OR “sugar substitute”[tiab] OR “Low-calorie sweeteners”[tiab] OR “artificial sugar”[tiab] OR “Steviol glycoside”[tiab] OR truvia[tiab] OR “Pure Via”[tiab] OR "Sweetening Agents"[Mesh] OR "Sweetening Agents"[tiab] OR "Sweetening "[tiab] OR acesulfame[tiab] OR sweeteners[tiab]) AND (trial*[title] OR “clinical trial”[tiab] OR “clinical trials”[tiab] OR “randomly assigned”[tiab] OR “random assignment”[tiab] OR Intervention*[tiab] OR randomiz*[tiab] OR randomis*[tiab] OR randomly[tiab] OR random*[tiab] OR assignment[tiab] OR "clinical trial"[publication type] OR “RCT”[tiab] OR "Clinical Trials as Topic"[Mesh] OR cross-over[tiab] OR parallel[tiab]) AND humans[MeSH] NOT (mouse[tiab] OR mice[mesh] OR "Rodentia"[Mesh] OR animal*[tiab] OR mice[tiab] OR mus[tiab] OR rat[tiab] OR rats[mesh] OR rats[tiab] OR swine[mesh] OR swine[tiab] OR dog [tiab] OR dogs[tiab] OR monkey[tiab] OR monkeys[tiab] OR monkies[tiab] OR cow[tiab] OR cows[tiab] OR hen[tiab] OR hens[tiab] OR pig[tiab] OR pigs[tiab] OR fish[tiab] OR fishes[tiab] OR cohort[tiab] OR "Cohort Studies"[Mesh] OR lamb[tiab] OR “in-vitro”[tiab] OR invitro[tiab] OR “in-vivo”[tiab] OR invivo[tiab] OR "in vitro"[tiab] OR "in vivo"[tiab] OR case-control[tiab] OR "Case-Control Studies"[Mesh] OR cross-sectional[tiab] OR "Cross-Sectional Studies"[Mesh] OR "Dietary Supplements"[Mesh] OR observational[title] OR "Observational Study" [Publication Type] OR "Observational Study" [Publication Type] OR "Monotremata"[Mesh] OR "Proboscidea Mammal"[Mesh] OR "Marsupialia"[Mesh] OR "Amphibians"[Mesh] OR "Birds"[Mesh] OR "Fishes"[Mesh] OR "Reptiles"[Mesh] OR Rabbit[tiab] OR Rooster[tiab] OR suide[tiab] OR broilers[tiab] OR broiler[tiab] OR bacter*[tiab] OR in-vitro[tiab] OR "in vitro"[tiab])) |
| --- |
| **Scopus**  (TITLE-ABS-KEY("Non-Nutritive Sweeteners”) OR TITLE-ABS-KEY("Non Nutritive Sweeteners") OR TITLE-ABS-KEY(Aspartame) OR TITLE-ABS-KEY(Stevia) OR TITLE-ABS-KEY(Saccharin) OR TITLE-ABS-KEY (acetosulfame) OR TITLE-ABS-KEY(acesulfame K) OR TITLE-ABS-KEY(acesulfame potassium) OR TITLE-ABS-KEY(“Ace-K”) OR TITLE-ABS-KEY(“AceK”) OR TITLE-ABS-KEY(Sorbitol) OR TITLE-ABS-KEY(trichlorosucrose) OR TITLE-ABS-KEY(sucralose) OR TITLE-ABS-KEY (“artificial sweeteners”) OR TITLE-ABS-KEY ("Carbonated Beverages") OR TITLE-ABS-KEY (“diet soda”) OR TITLE-ABS-KEY(“Sugary Soda”) OR TITLE-ABS-KEY(“Sugar Sweetened Soda”) OR TITLE-ABS-KEY (“Artificially Sweetened Soda”) OR TITLE-ABS-KEY(“non-caloric sweeteners”) OR TITLE-ABS-KEY (“non caloric sweeteners”) OR TITLE-ABS-KEY(Advantame) OR TITLE-ABS-KEY(Neotame) OR TITLE-ABS-KEY(“zero-calorie sweetener”) OR TITLE-ABS-KEY(NutraSweet) OR TITLE-ABS-KEY(Splenda) OR TITLE-ABS-KEY(Cyclamates) OR TITLE-ABS-KEY(cyclamic acid) OR TITLE-ABS-KEY("acetosulfame") OR TITLE-ABS-KEY(“high-intensity sweetener”) OR TITLE-ABS-KEY(“Steviol glycosides”) OR TITLE-ABS-KEY("rebaudioside A") OR TITLE-ABS-KEY(“reb a”) OR TITLE-ABS-KEY(stevioside) OR‌ TITLE-ABS-KEY("rebaudioside D" ) OR TITLE-ABS-KEY(newtame) OR TITLE-ABS-KEY("sugar twin") OR TITLE-ABS-KEY(nutrasweet ) OR TITLE-ABS-KEY(“necta sweet”) OR TITLE-ABS-KEY(“sweet’n low”) OR TITLE-ABS-KEY(“Luo Han Guo”) OR TITLE-ABS-KEY(“monk fruit”) OR TITLE-ABS-KEY(“Siraitia grosvenorii Swingle fruit extract”) OR TITLE-ABS-KEY(“sugar substitute”) OR TITLE-ABS-KEY(“Low-calorie sweeteners”) OR TITLE-ABS-KEY(“artificial sugar”) OR TITLE-ABS-KEY(“Steviol glycoside”) OR TITLE-ABS-KEY(truvia) OR TITLE-ABS-KEY(“Pure Via”) OR TITLE-ABS-KEY("Sweetening Agents”) OR TITLE-ABS-KEY(Sweetening) OR TITLE-ABS-KEY(acesulfame) OR TITLE-ABS-KEY(sweeteners)) AND (TITLE(trial*) OR TITLE-ABS-KEY(“clinical trial”) OR TITLE-ABS-KEY(“clinical trials”) OR TITLE-ABS-KEY ("randomly assigned") OR TITLE-ABS-KEY("random assignment") OR TITLE-ABS-KEY(Intervention*) OR TITLE-ABS-KEY(randomiz*) OR TITLE-ABS-KEY(randomis*) OR TITLE-ABS-KEY(randomly) OR TITLE-ABS-KEY(assignment) OR TITLE-ABS-KEY(RCT) OR TITLE-ABS-KEY(cross-over) OR TITLE-ABS-KEY(parallel) OR TITLE-ABS-KEY(random*)) AND NOT (TITLE(observational) OR TITLE-ABS-KEY(mouse) OR TITLE-ABS-KEY(mice) OR TITLE-ABS-KEY(rat) OR TITLE-ABS-KEY(rats) OR TITLE-ABS-KEY(dog) OR TITLE-ABS-KEY(dogs) OR TITLE-ABS-KEY(monkey) OR TITLE-ABS-KEY(monkeys) OR TITLE-ABS-KEY(monkies) OR TITLE-ABS-KEY(cohort) OR TITLE-ABS-KEY("Cohort Studies") OR TITLE-ABS-KEY(case-control) OR TITLE-ABS-KEY("Case-Control Studies") OR TITLE-ABS-KEY(cross-sectional) OR TITLE-ABS-KEY("Cross-Sectional Studies") OR TITLE-ABS-KEY(cow) OR TITLE-ABS-KEY(cows) OR TITLE-ABS-KEY(hen) OR TITLE-ABS-KEY(hens) OR TITLE-ABS-KEY(pig) OR TITLE-ABS-KEY(pigs) OR TITLE-ABS-KEY(fish) OR TITLE-ABS-KEY(fishes) OR TITLE-ABS-KEY(animal*) OR TITLE-ABS-KEY(lamb) OR TITLE-ABS-KEY("in-vitro") OR TITLE-ABS-KEY(invitro) OR TITLE-ABS-KEY("in-vivo") OR TITLE-ABS-KEY(invivo) OR TITLE-ABS-KEY("in vitro") OR TITLE-ABS-KEY("in vivo") OR TITLE-ABS-KEY("observational study") OR TITLE-ABS-KEY("observational studies") OR TITLE-ABS-KEY(Monotremata) OR TITLE-ABS-KEY("Proboscidea Mammal") OR TITLE-ABS-KEY("Marsupialia") OR TITLE-ABS-KEY(Amphibians) OR TITLE-ABS-KEY(Bird) OR TITLE-ABS-KEY(birds) OR TITLE-ABS-KEY(Reptiles) OR TITLE-ABS-KEY(Rabbit) OR TITLE-ABS-KEY(Rooster) OR TITLE-ABS-KEY(suide) OR TITLE-ABS-KEY(broilers) OR TITLE-ABS-KEY(broiler) OR TITLE-ABS-KEY(bacter*) OR TITLE-ABS-KEY(mus) OR TITLE-ABS-KEY(swine) OR TITLE-ABS-KEY(rodent)) |
| **ISI Web of Science**  (TS= ("Non-Nutritive Sweeteners”) OR TS= ("Non Nutritive Sweeteners”) OR TS= (Aspartame) OR TS= (Stevia) OR TS= (Saccharin) OR TS= (acetosulfame) OR TS= (acesulfame K) OR TS= (acesulfame potassium) OR TS= (“Ace-K”) OR TS= (“AceK”) OR TS= (Sorbitol) OR TS= (trichlorosucrose) OR TS= (sucralose ) OR TS= (“artificial sweeteners”) OR TS= ("Carbonated Beverages") OR TS= (“diet soda”) OR TS= (“Sugary Soda”) OR TS= (“Sugar Sweetened Soda”) OR TS= (“Artificially Sweetened Soda”) OR TS= (“non-caloric sweeteners”) OR TS= (“non caloric sweeteners”) OR TS= (Advantame) OR TS= (Neotame) OR TS= (“zero-calorie sweetener”) OR TS= (NutraSweet) OR TS= (Splenda) OR TS= (Cyclamates) OR TS= (cyclamic acid) OR TS= ("acetosulfame") OR TS= (“high-intensity sweetener”) OR TS= (“Steviol glycosides”) OR TS= ("rebaudioside A") OR TS= (“reb a”) OR TS= (stevioside) OR TS= ("rebaudioside D" ) OR TS= (newtame) OR TS= ("sugar twin") OR TS= (nutrasweet ) OR TS= (“necta sweet”) OR TS= (“sweet’n low”) OR TS= (“Luo Han Guo”) OR TS= (“monk fruit”) OR TS= (“Siraitia grosvenorii Swingle fruit extract”) OR‌ TS= (“sugar substitute”) OR TS= (“Low-calorie sweeteners”) OR TS= (“artificial sugar”) OR TS= (“Steviol glycoside”) OR TS= (truvia) OR TS= (“Pure Via”) OR TS=("Sweetening Agents”) OR TS= (Sweetening) OR TS= (acesulfame) OR TS=(sweeteners)) AND (TI=(trial*) OR TS=(“clinical trial”) OR TS=(“clinical trials”) OR TS= ("randomly assigned") OR TS=("random assignment") OR TS=(Intervention*) OR TS=(randomize*) OR TS=(randomis*) OR TS=(randomly) OR TS=(assignment) OR TS=(RCT) OR TS=(cross-over) OR TS=(parallel) OR TS=( random*)) NOT (TI=(observational) OR TS=(mouse) OR TS=(mice) OR TS=(rat) OR TS=(rats) OR TS=(dog) OR TS=(dogs) OR TS=(monkey) OR TS=(monkeys) OR TS=(monkies) OR TS=(cow) OR TS=(cows) OR TS=(hen) OR TS=(hens) OR TS=(pig) OR TS=(pigs) OR TS=(fish) OR TS=(fishes) OR TS=(animal*) OR TS=(lamb) OR TS=("in-vitro") OR TS=(invitro) OR TS=("in-vivo") OR TS=(invivo) OR TS=("in vitro") OR TS=("in vivo") OR TS=(cohort) OR TS= ("Cohort Studies”) OR TS= (case-control) OR TS= ("Case-Control Studies") OR TS= (cross-sectional) OR TS= ("Cross-Sectional Studies") OR TS=("observational study") OR TS=("observational studies") OR TS=(Monotremata) OR TS=("Proboscidea Mammal") OR TS=("Marsupialia") OR TS=(Amphibians) OR TS=(Bird) OR TS=(birds) OR TS=(Reptiles) OR TS=(Rabbit) OR TS=(Rooster) OR TS=(suide) OR TS=(broilers) OR TS=(broiler) OR TS=(*bacter) OR TS= (bacter*) OR TS=(mus) OR TS=(swine) OR TS= (rodent)) |

**Legends to supplementary Figures**

**Supplemental** **Figure 1.** Begg’s Funnel plot depicting effect sizes against their standard error for the effect of NNSs consumption vs sugar on total energy intake

**Supplemental Figure 2.** Begg’s Funnel plot depicting effect sizes against their standard error for the effect of NNSs consumption vs water on total energy intake

**Supplemental Figure 3.** Begg’s Funnel plot depicting effect sizes against their standard error for the effect of NNSs consumption vs sugar on carbohydrate intake

**Supplemental Figure 4.** Begg’s Funnel plot depicting effect sizes against their standard error for the effect of NNSs consumption vs water on carbohydrate intake

**Supplemental Figure 5.** Begg’s Funnel plot depicting effect sizes against their standard error for the effect of NNSs consumption vs sugar on fat intake

**Supplemental Figure 6.** Begg’s Funnel plot depicting effect sizes against their standard error for the effect of NNSs consumption vs water on fat intake

**Supplemental Figure 7.** Begg’s Funnel plot depicting effect sizes against their standard error for the effect of NNSs consumption vs sugar on protein intake

**Supplemental Figure 8.** Begg’s Funnel plot depicting effect sizes against their standard error for the effect of NNSs consumption vs water on protein intake

**Supplemental Figure 9.** Begg’s Funnel plot depicting effect sizes against their standard error for the effect of NNSs consumption vs sugar on sugar intake

**Supplemental Figure 10.** Begg’s Funnel plot depicting effect sizes against their standard error for the effect of NNSs consumption vs water on sugar intake

**Supplemental Figure 11.** Begg’s Funnel plot depicting effect sizes against their standard error for the effect of NNSs consumption vs sugar on fiber intake

**Supplemental Figure 12.** Begg’s Funnel plot depicting effect sizes against their standard error for the effect of NNSs consumption vs water on fiber intake

**Supplemental Figure1**


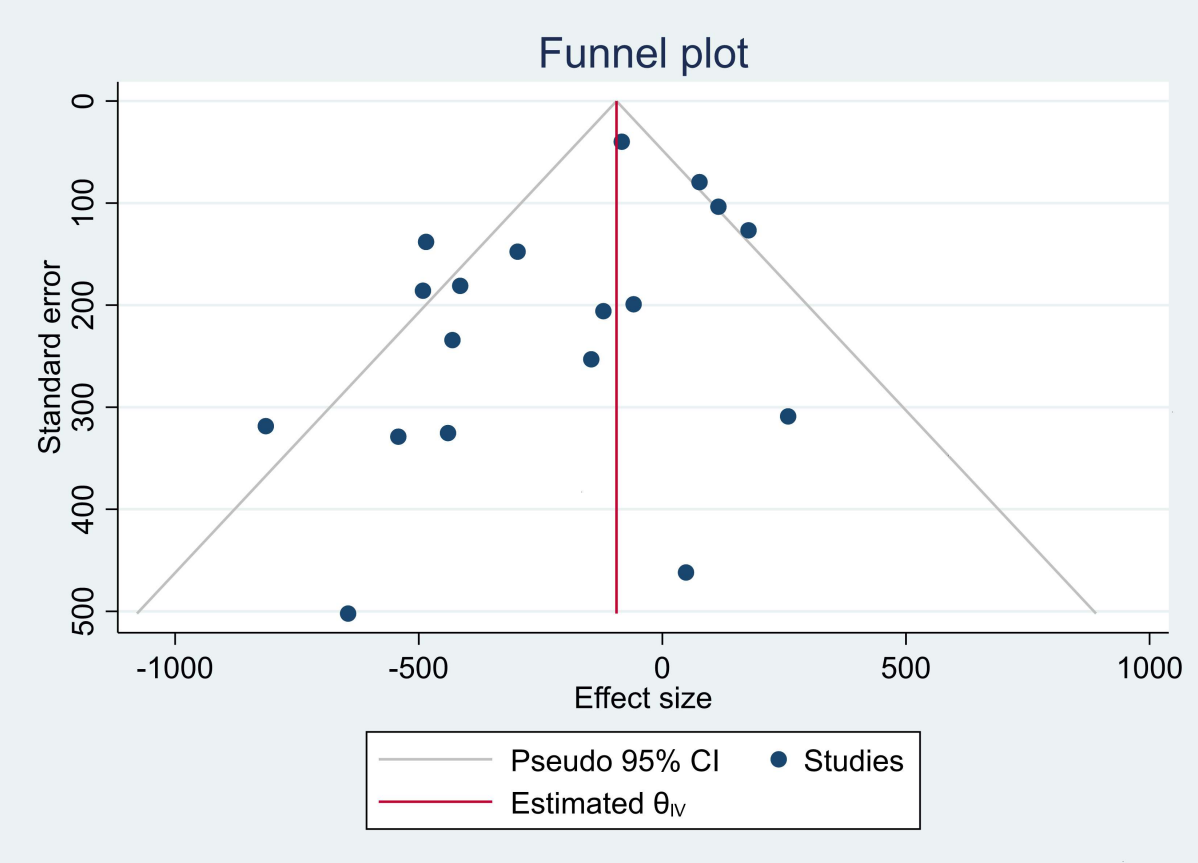


**Supplemental Figure 2**


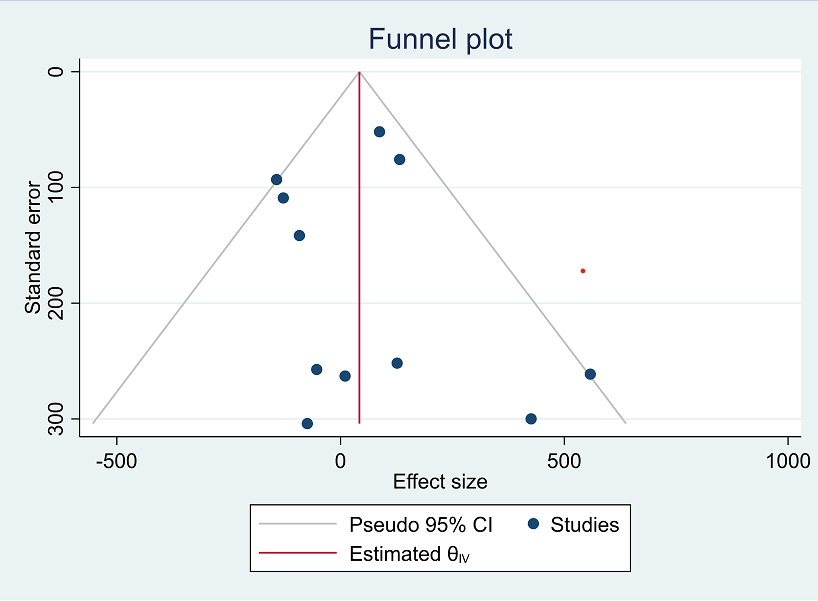


**Supplemental Figure 3**


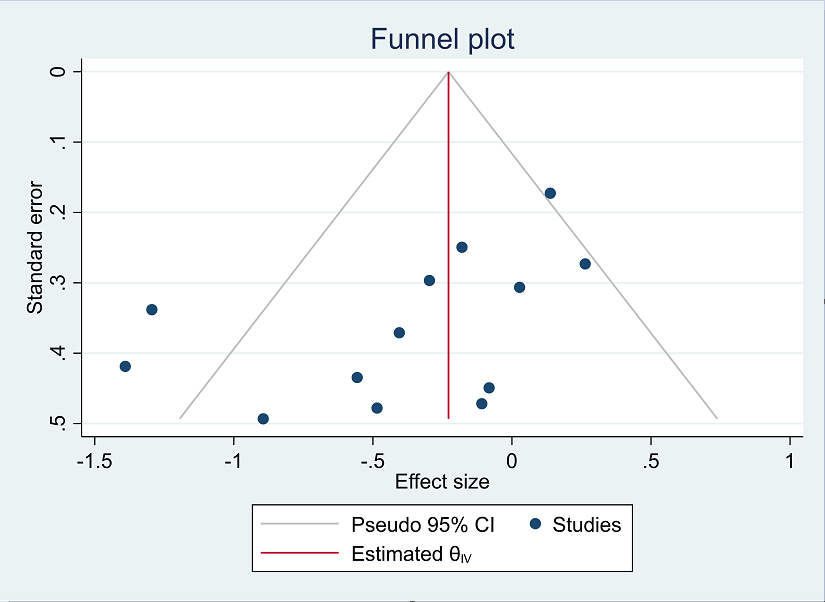


**Supplemental Figure 4**


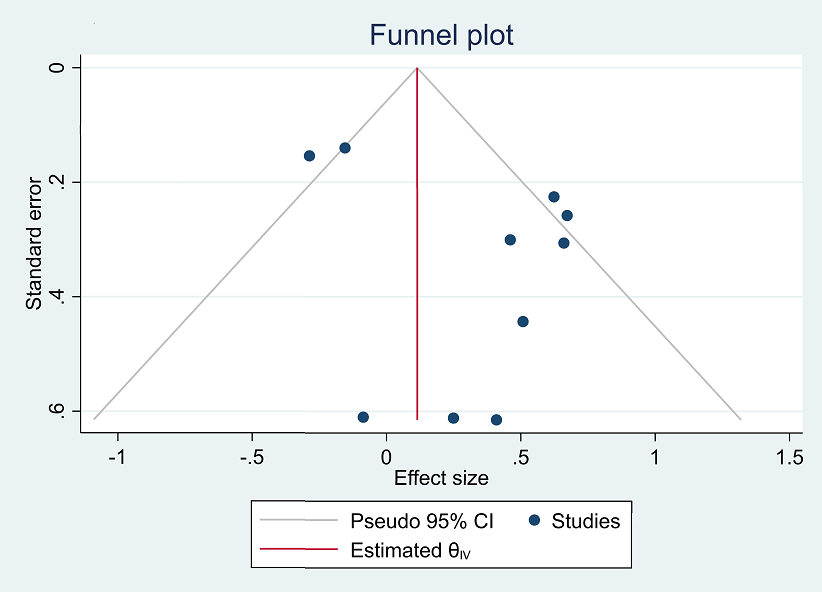


**Supplemental Figure 5**

**
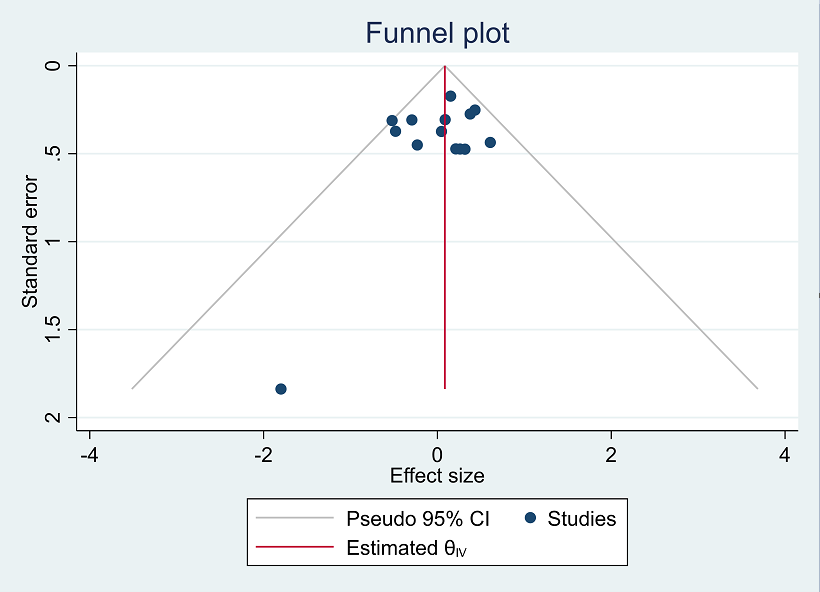
**

**Supplemental Figure 6**

**Supplemental Figure 7**


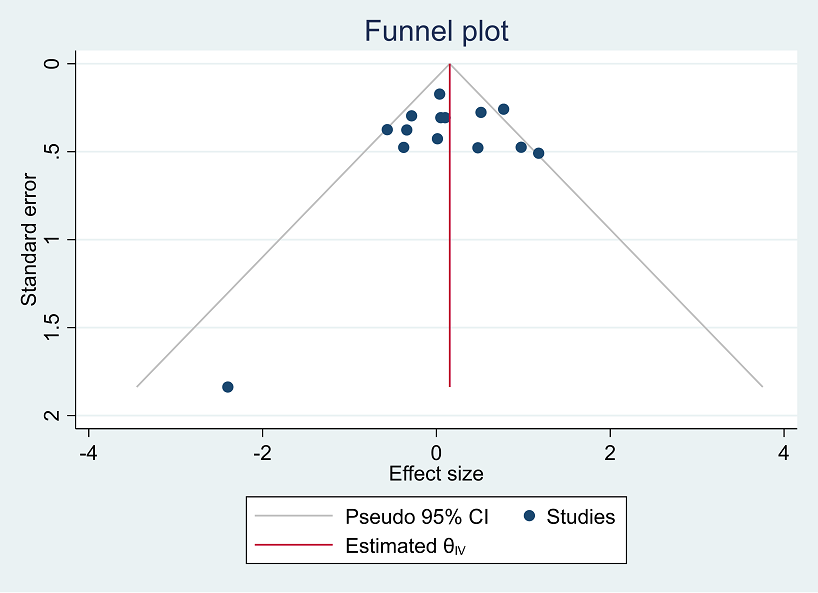


**Supplemental Figure 8**


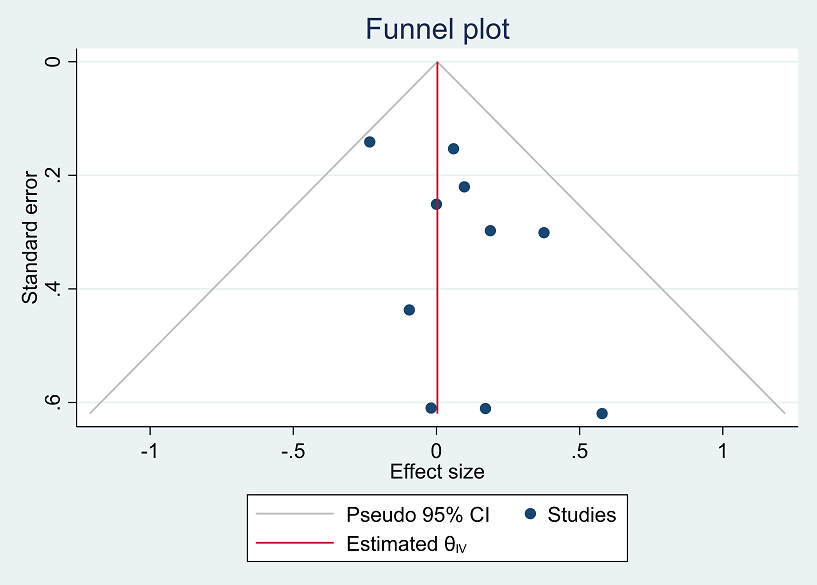


**Supplemental Figure 9**


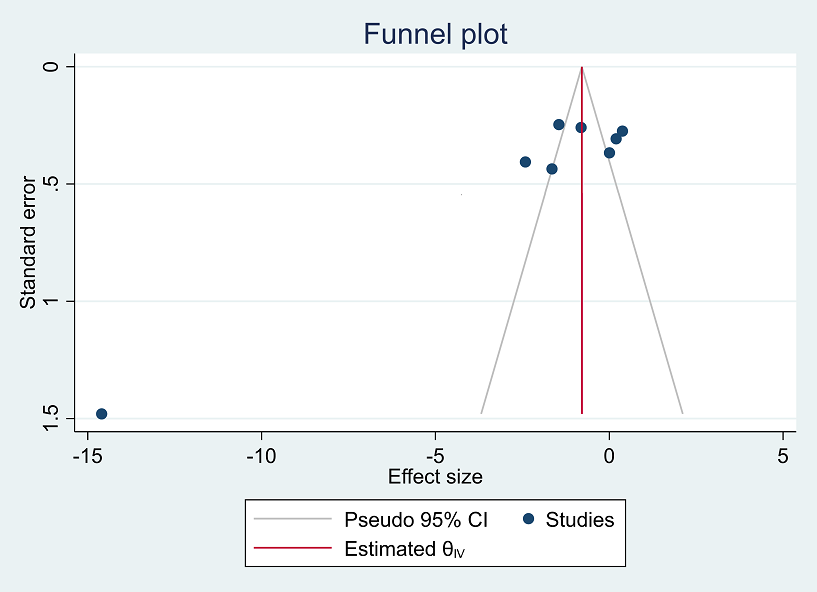


**Supplemental Figure 10**

**Supplemental Figure 11**

**Supplemental Figure 12**
